# Association between the triglyceride-glucose index and functional outcome at discharge in patients receiving standardized treatment for atherosclerotic and cardioembolic stroke

**DOI:** 10.3389/fneur.2026.1899230

**Published:** 2026-10-01

**Authors:** Nini Zhu, Wen Zhong, Xiaozhu Shen, Jingxian Liao, Guanghui Zhang, Xiguang Liu

**Affiliations:** 1 School of Medicine, Jiangsu University, Zhenjiang, China; 2 Department of Geriatrics, Lianyungang Second People’s Hospital, Lianyungang, China; 3 Department of Neurology, The Affiliated Lianyungang Hospital of Xuzhou Medical University, Lianyungang, China; 4 Department of Neurosurgery, Lianyungang First People’s Hospital, Lianyungang, China

**Keywords:** atherosclerotic stroke, cardioembolic stroke, prognosis, standardized treatment, triglyceride-glucose index

## Abstract

**Objective:**

The triglyceride-glucose (TyG) index is gaining considerable attention as an emerging marker of metabolic syndrome. The TyG index is strongly associated with atherosclerosis and cardiovascular disease development. However, the impact of the TyG index on the functional outcome at discharge of patients receiving standardized treatment and its variability in different types of stroke have not been fully elucidated.

**Method:**

Baseline data, neurological scale scores, and laboratory indicators were retrospectively collected from ischemic stroke patients at Lianyungang Second People’s Hospital and Lianyungang First People’s Hospital. Patients were categorized into good and poor functional outcome groups based on the modified Rankin Scale (mRS) score at discharge. A logistic regression model was used to evaluate the relationship between the TyG index and functional outcome at discharge of patients with atherosclerotic and cardioembolic stroke.

**Results:**

A total of 854 patients were included in the analysis, including 712 patients with large artery atherosclerotic (LAA) and 142 patients with cardioembolism (CE). Subgroup comparisons based on different discharge-level functional outcomes showed that the TyG index of the 187 patients in the poor functional outcome at discharge group was higher than that of patients in the good functional outcome group (8.98 vs. 8.69). The restricted cubic curve revealed a nonlinear association between the TyG index and discharge-level functional outcome among LAA stroke patients (for nonlinearity, *p* = 0.026). Multivariate logistic regression showed that the TyG-Q2 and TyG-Q3 groups were not associated with discharge-level functional outcome compared with the TyG-Q1 group, and the TyG-Q4 group was a risk factor for the occurrence of poor functional in the LAA group (odds ratio (OR) = 2.63, 95% confidence interval (CI) = 1.52–4.55). After adjusting for age, National Institute of Health Stroke Scale (NIHSS) score at admission, glucose, diastolic blood pressure, uric acid, and low-density lipoprotein, the TyG-Q4 group remained a risk factor for the occurrence of poor functional outcome at discharge in the LAA group (OR = 2.38, 95% CI = 1.03–5.51). However, the TyG index did not correlate with functional outcome at discharge in the CE group. Of note, this negative CE subgroup result should be interpreted cautiously due to the limited sample size and insufficient endpoint events, which may cause inadequate statistical power and false-negative outcomes; thus, the potential predictive value of the TyG index for discharge-level functional outcome in CE stroke cannot be excluded.

**Conclusion:**

The TyG index was identified as a risk factor for poor functional outcome at discharge in LAA stroke but was not correlated with the functional outcome at discharge of CE stroke. Considering the limited sample size and insufficient statistical power of the CE subgroup, this negative result should not be over-interpreted, and large-scale clinical studies are required to further explore the potential prognostic value of the TyG index in cardioembolic stroke.

## Introduction

Stroke is one of the leading causes of death and long-term disability worldwide, imposing significant social and economic burdens (1–3). Stroke is primarily classified into hemorrhagic and ischemic types, with ischemic stroke accounting for the majority of cases. According to the 1993 Trial of ORG 10172 in Acute Stroke Treatment (TOAST) classification, ischemic stroke is divided into five categories: large-artery atherosclerosis (LAA), cardioembolism (CE), small-artery occlusion, stroke of other determined etiology, and stroke of undetermined etiology (4, 5). LAA is primarily caused by the rupture of atherosclerotic plaques or thrombus formation in large arteries, leading to blood flow obstruction and ultimately cerebral infarction (6). CE is mainly caused by emboli from heart diseases, such as atrial fibrillation, which dislodge and enter the cerebral blood vessels, causing impaired cerebral blood flow (7). Despite the differences in their pathological mechanisms, both LAA and CE can result in severe neurological deficits and may even be life-threatening.

Conservative treatment is recommended for patients with ischemic stroke if the window for thrombolysis or thrombectomy has passed (8). This treatment primarily includes antiplatelet drugs, anticoagulants, lipid-lowering medications, and comprehensive management of risk factors such as hypertension and diabetes (8, 9). These measures aim to stabilize clinical status, lower the risk of recurrent vascular events, and optimize patient functional recovery. However, responses to conservative treatment vary significantly among patients, complicating outcome prediction.

The triglyceride-glucose (TyG) index, a metabolic marker reflecting insulin resistance, has gradually attracted widespread research attention in recent years (10, 11). Insulin resistance is a core feature of metabolic syndrome and is closely associated with the occurrence of atherosclerosis and cardiovascular diseases (12). The TyG index, calculated using fasting triglycerides (TGs) and fasting glucose (Glu), is simple and practical. It is significantly associated with the development of various metabolic-related diseases, such as atherosclerotic cardiovascular diseases and type 2 diabetes (13, 14). Some studies have also suggested that the TyG index can serve as an effective indicator for predicting the progression of atherosclerosis and the risk of cardiovascular events (15). However, although the predictive value of the TyG index in cardiovascular and metabolic diseases is increasingly recognized, its clinical application in stroke, especially among different types of stroke, remains limited (13). Whether the TyG index can predict functional outcome at discharge following conservative treatment has not been fully compared between atherosclerotic and cardioembolic stroke subtypes. Therefore, investigating the role of the TyG index in stroke patients, particularly its predictive performance across different stroke subtypes, is of great clinical significance.

The present study sought to investigate the association between the TyG index and functional outcome at discharge of patients with atherosclerotic and cardioembolic stroke receiving conservative treatment. The admission-level TyG index was calculated through retrospective analysis of data from patients with atherosclerotic or cardioembolic stroke, and its association with discharge-level functional outcome was evaluated. This study aims to explore the predictive performance of the TyG index across different stroke subtypes and provide reference information for risk-stratified clinical decision-making and patient management.

## Methods

### Study design and population

Clinical data were retrospectively collected from 444 ischemic stroke patients admitted at Lianyungang Second People’s Hospital between January 1, 2022 and July 31, 2024 and from 410 ischemic stroke patients admitted at Lianyungang First People’s Hospital between October 2019 and December 2021. The flowchart of the patient selection process is presented in Figure 1. This study was approved by the ethics committees of Lianyungang Second People’s Hospital and Lianyungang First People’s Hospital. All protocols and procedures were conducted following the Declaration of Helsinki. Informed consent was obtained from all subjects.

**Figure 1:**
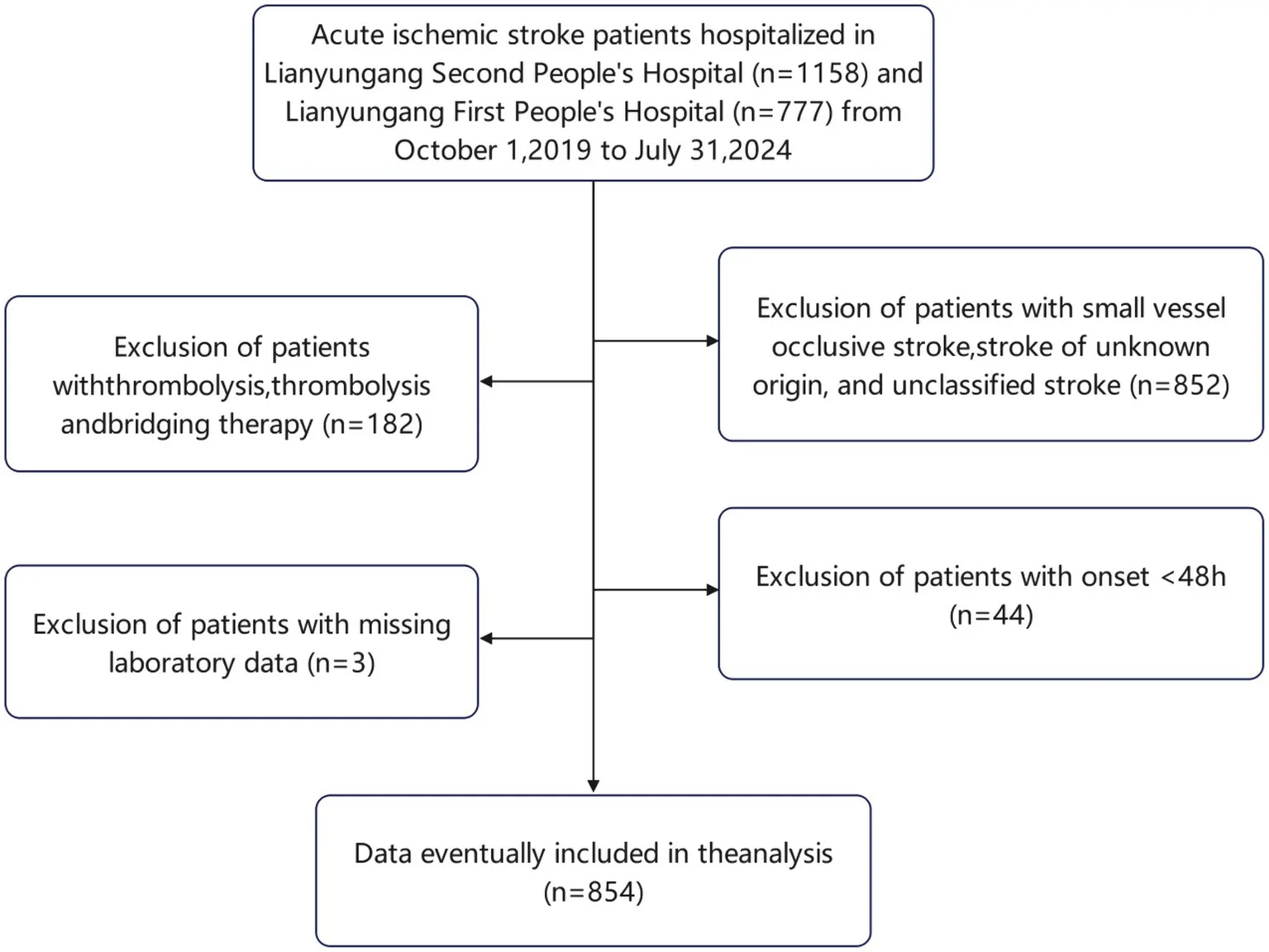
Flowchart of study participants.

Inclusion criteria: (1) patients aged ≥18 years; (2) those who met the diagnostic criteria for acute ischemic stroke; (3) stroke onset >48 h prior; (4) those receiving standardized medical treatment; and (5) those who participated voluntarily and provided informed consent.

Exclusion criteria: (1) patients with intracranial hemorrhage or mass; (2) those receiving thrombolysis or thrombectomy; (3) those with severe infection or septic shock; (4) those with liver or kidney failure; (5) those with incomplete laboratory or clinical data; and (6) those with small vessel occlusion stroke, stroke of unknown cause, and unclassified stroke based on the TOAST classification.

### Data collection

Baseline data for all enrolled patients were collected from the hospital database, including age, gender, systolic blood pressure, diastolic blood pressure, and medical history, including a history of hypertension, diabetes, smoking, and alcohol consumption. Blood samples were collected within 24 h of admission by trained medical staff for laboratory testing. Laboratory tests included creatinine (Cr), homocysteine (Hcy), total cholesterol (TC), TGs, low-density lipoprotein (LDL), high-density lipoprotein (HDL), glucose (Glu), glycated hemoglobin (HbA1c), uric acid (UA), and blood urea nitrogen (BUN). National Institute of Health Stroke Scale (NIHSS) and modified Rankin Scale (mRS) scores were assessed by two experienced neurologists at both admission and discharge. Patients were classified into the LAA and CE groups according to TOAST. Stroke severity was assessed using the NIHSS score, with ≤4 indicating mild stroke and >4 indicating severe stroke.

### Assessment of disease functional outcome at discharge

Two experienced neurologists assessed functional outcomes at discharge using the mRS, with an mRS score of 0–3 indicating a good outcome, and mRS > 3 defined as poor functional outcome at hospital discharge. In-hospital death (mRS = 6) was included within the poor-outcome group. Pre-stroke mRS was abstracted from medical records for all enrolled patients and considered in our analyses.

### Standardized treatment protocols

All enrolled patients received standardized comprehensive treatment strictly based on the latest Chinese Guidelines for the Diagnosis and Treatment of Acute Ischemic Stroke. The detailed treatment regimens were uniformly implemented in our two study centers to minimize treatment heterogeneity. Specifically, individualized anti-ischemic, lipid-regulating, blood pressure and glucose management, and early rehabilitation strategies were applied according to stroke subtype and personal baseline conditions. For antiplatelet and anticoagulation therapy, patients diagnosed with LAA stroke received single or dual antiplatelet therapy tailored to vascular stenosis severity and plaque stability, while all CE stroke patients underwent standardized anticoagulation treatment to prevent cardioembolic thrombus recurrence. For lipid management, high-intensity statin therapy was routinely administered during hospitalization to stabilize atherosclerotic plaques and reduce lipid levels. Individualized antihypertensive and hypoglycemic treatments were adopted throughout hospitalization to maintain stable blood pressure and blood glucose levels and avoid hemodynamic or metabolic fluctuations. In addition, all patients received standardized early rehabilitation training, including physical exercise and neurological function rehabilitation, to promote the recovery of neurological deficits.

### Assessment of the TyG index

The TyG index was calculated as the natural logarithm (Ln) of the product of blood Glu and TG using the formula: Ln[fasting TGs (mg/dL) × fasting blood Glu (mg/dL)/2] and categorized into quartiles as follows: Q1, <8.32; Q2, 8.32–8.74; Q3, 8.75–9.31; and Q4, >9.32.

### Statistical analysis

Statistical analysis was performed using R version 4.3.2 (R Core Team 2022). All statistical tests were two-sided, and *p* < 0.05 was considered statistically significant. The Kolmogorov–Smirnov test was used to assess the normality of the data. Normally distributed continuous data were expressed as mean ± standard deviation, while skewed data were presented as median and interquartile range (P25–P75). Categorical data were expressed as percentages (%). Normally distributed data were compared using analysis of variance (ANOVA), and non-normally distributed data were analyzed using the Kruskal-Wallis test. Fisher’s exact test or a Chi-squared test was used as appropriate to compare categorical variables. Variables with >10% missing data were excluded, and imputation was performed for variables with <10% missing data using the random forest method in the R package “mice.”

Meanwhile, to eliminate multicollinearity interference and reduce residual confounding bias in variable screening, variance inflation factors (VIFs) and tolerance values of all candidate variables were strictly calculated and evaluated in this study. A rigorous multicollinearity screening criterion was adopted: variables with a VIF > 5 were considered to have significant multicollinearity and were excluded from the final regression model, while variables with VIF ≤ 5 were retained for subsequent confounding adjustment and statistical analysis. Restricted cubic spline (RCS) regression was used to explore potential nonlinear associations between the TyG index and functional outcome at discharge in LAA and CE patients while adjusting for confounding factors such as gender, age, history of hypertension, diabetes, smoking, and drinking, NIHSS score at admission, diastolic blood pressure (DBP), Glu, UA, and LDL.

Univariate logistic regression analysis was used to investigate the association between baseline information and functional outcome at discharge. A multivariate logistic regression model was employed to determine the relationship between the TyG index and functional outcomes in LAA and CE patients while adjusting for confounding factors. Three multivariate logistic regression models were established. The prognostic value of the TyG index in patients with CE and LAA was evaluated using receiver operating characteristic (ROC) curves.

To test the robustness and potential variations in different subgroups, stratified analyses were performed based on gender (male and female), age group (<60 years and ≥60 years, with ≥60 years being considered elderly by the World Health Organization (16)), history of diabetes, hypertension, smoking, and drinking, and NIHSS score at admission (≤4 for mild stroke, >4 for severe stroke).

## Results

### Baseline characteristics of patients based on TyG index quartiles

A total of 854 patients were included in the analysis and divided into four groups based on TyG index quartiles: Q1, Q2, Q3, and Q4 groups. Table 1 summarizes the clinical characteristics of all patients. The proportion of LAA patients was higher than that of CE in the four groups (*p* < 0.001). No statistically significant differences were found among the four groups regarding NIHSS scores at admission, systolic blood pressure (SBP), BUN, smoking, and drinking. However, statistically significant differences were observed among the four groups in terms of age, gender, history of diabetes and hypertension, DBP, Hcy, HbA1c, Cr, Glu, TG, TC, HDL, and LDL levels.

**Table 1:** Characteristics of study participants according to TyG index quartiles.

Characteristic	TyG index quartiles				*p*-value
	Q1	Q2	Q3	Q4	
Age, *M* (Q1, Q3) (years)	72 (65, 81)	70 (63, 77)	68 (61, 75)	66 (58, 74)	<0.001^***^
Gender, male, *n* (%)	144 (67.9%)	148 (69.2%)	123 (57.7%)	117 (54.4%)	0.002^**^
Smoking, *n* (%)	62 (29.2%)	63 (29.4%)	61 (28.6%)	56 (26.0%)	0.854
Drinking, *n* (%)	43 (20.3%)	53 (24.8%)	45 (21.1%)	47 (21.9%)	0.702
Diabetes, *n* (%)	23 (10.8%)	49 (22.9%)	82 (38.5%)	111 (51.6%)	<0.001^***^
Hypertension, *n* (%)	131 (61.8%)	146 (68.2%)	161 (75.6%)	172 (80.0%)	<0.001^***^
SBP, *M* (Q1, Q3) (mmHg)	148 (135, 159)	150 (136, 162)	149 (138, 160)	151 (140, 166)	0.052
DBP, *M* (Q1, Q3) (mmHg)	85 (77, 96)	85 (78, 95)	86 (80, 98)	90 (80, 100)	0.005^**^
HbA1c, *M* (Q1, Q3) (%)	5.70 (5.50, 6.00)	5.90 (5.60, 6.30)	6.40 (5.90, 7.70)	8.00 (6.10, 9.30)	<0.001^***^
Glu, *M* (Q1, Q3) (mmol/L)	4.96 (4.57, 5.38)	5.43 (4.94, 6.12)	6.19 (5.48, 7.87)	8.87 (6.42, 11.59)	<0.001^***^
TC, *M* (Q1, Q3) (mmol/L)	4.06 (3.45, 4.58)	4.38 (3.80, 4.99)	4.72 (4.02, 5.51)	4.91 (4.13, 5.72)	<0.001^***^
TG, *M* (Q1, Q3) (mmol/L)	0.79 (0.65, 0.92)	1.17 (1.03, 1.30)	1.58 (1.25, 1.89)	2.68 (1.95, 3.70)	<0.001^***^
HDL, *M* (Q1, Q3) (mmol/L)	1.12 (0.99, 1.34)	1.09 (0.93, 1.21)	1.09 (0.96, 1.24)	1.07 (0.93, 1.21)	0.012^*^
LDL, *M* (Q1, Q3) (mmol/L)	2.33 (1.89, 2.66)	2.62 (2.15, 3.10)	2.84 (2.29, 3.36)	2.83 (2.35, 3.44)	<0.001^***^
Hcy, *M* (Q1, Q3) (μmol/L)	11.6 (9.7, 14.5)	11.5 (9.0, 14.3)	10.2 (8.4, 12.3)	9.7 (7.8, 12.3)	<0.001^***^
BUN, *M* (Q1, Q3) (mmol/L)	6.10 (5.09, 7.47)	5.83 (4.90, 6.65)	5.77 (5.00, 7.10)	5.89 (4.65, 7.11)	0.159
Cr, *M* (Q1, Q3) (μmol/l)	67 (57, 81)	64 (54, 79)	62 (52, 77)	60 (48, 73)	<0.001^***^
UA, *M* (Q1, Q3) (μmol/l)	313 (266, 371)	331 (263, 389)	309 (254, 370)	293 (244, 365)	0.026^*^
TOAST, *n* (%)					0.041^*^
LAA	164 (77.4%)	186 (86.9%)	178 (83.6%)	184 (85.6%)	
CE	48 (22.6%)	28 (13.1%)	35 (16.4%)	31 (14.4%)	
NIHSS score at admission, *M* (Q1, Q3)	3.0 (1.0, 7.0)	3.0 (1.0, 6.0)	3.0 (1.0, 6.0)	4.0 (1.5, 8.0)	0.193
NIHSS score at discharge, *M* (Q1, Q3)	2.0 (1.0, 5.0)	2.0 (1.0, 4.0)	2.0 (1.0, 5.0)	2.0 (1.0, 7.0)	0.033^*^
mRS score at admission, *M* (Q1, Q3)	3.00 (1.00, 4.00)	3.00 (1.00, 4.00)	3.00 (2.00, 4.00)	3.00 (2.00, 4.00)	0.067
mRS score at discharge, *M* (Q1, Q3)	1.00 (1.00, 3.00)	1.00 (1.00, 3.00)	2.00 (1.00, 3.00)	2.00 (1.00, 4.00)	0.005^**^
mRS score at discharge					<0.001^***^
≤3	169 (79.7%)	182 (85.0%)	170 (79.8%)	146 (67.9%)	
>3	43 (20.3%)	32 (15.0%)	43 (20.2%)	69 (32.1%)	

SBP, systolic blood pressure; DBP, diastolic blood pressure; Hcy, homocysteine; HbA1c, glycosylated hemoglobin; Cr, creatinine; Glu, glucose; TC, cholesterol; TG, triglyceride; HDL, high density lipoprotein; LDL, low-density lipoprotein; TOAST, trial of org 10172 in acute stroke treatment; CE, cardioembolic; LAA, large artery atherosclerotic; NIHSS, National Institute of Health Stroke Scale; mRS, modified Rankin Scale; *p < 0.05; **p < 0.01; ***p < 0.001.

A comparison based on different discharge-level functional outcomes is presented in Table 2. Of the 854 patients, 187 had poor functional outcome at discharge, and the TyG index in the poor functional outcome at discharge group was higher than that in the good functional outcome group (8.98 vs. 8.69). However, no statistically significant differences were observed between the two groups regarding SBP, DBP, TG, HDL, Hcy, BUN, Cr, and history of smoking, drinking, diabetes, and hypertension.

**Table 2:** Baseline characteristics of LAA and CE compared according to prognosis.

Characteristic	LAA			CE		
	Excellent prognosis	Poor prognosis	*p*-value	Excellent prognosis	Poor prognosis	*p*-value
Age, *M* (Q1, Q3) (years)	68 (60, 75)	70 (63, 78)	0.026^*^	71 (63, 78)	80 (69, 84)	<0.001^***^
Gender, male, *n* (%)	365 (63.8%)	81 (57.9%)	0.192	64 (67.4%)	22 (46.8%)	0.018^*^
Smoking, *n* (%)	164 (28.7%)	38 (27.1%)	0.719	32 (33.7%)	8 (17.0%)	0.038^*^
Drinking, *n* (%)	128 (22.4%)	29 (20.7%)	0.670	20 (21.1%)	11 (23.4%)	0.750
Hypertension, *n* (%)	420 (73.4%)	93 (66.4%)	0.098	63 (66.3%)	34 (72.3%)	0.468
Diabetes, *n* (%)	178 (31.1%)	50 (35.7%)	0.296	29 (30.5%)	8 (17.0%)	0.084
SBP, *M* (Q1, Q3) (mmHg)	150 (138, 161)	152 (137, 163)	0.567	146 (136, 158)	148 (138, 164)	0.366
DBP, *M* (Q1, Q3) (mmHg)	87 (79, 98)	82 (78, 93)	0.020^*^	86 (79, 95)	90 (80, 100)	0.176
HbA1c, *M* (Q1, Q3) (%)	6.00 (5.70, 7.40)	6.20 (5.65, 7.93)	0.274	6.10 (5.70, 7.00)	6.10 (5.70, 6.70)	0.905
Glu, *M* (Q1, Q3) (mmol/L)	5.59 (4.95, 7.22)	6.51 (5.29, 9.70)	<0.001^***^	5.53 (4.80, 6.51)	6.36 (5.76, 7.37)	<0.001^***^
TC, *M* (Q1, Q3) (mmol/L)	4.56 (3.87, 5.33)	4.38 (3.68, 4.99)	0.042^*^	4.35 (3.67, 4.94)	4.25 (3.63, 4.73)	0.668
TG, *M* (Q1, Q3) (mmol/L)	1.27 (0.95, 1.91)	1.41 (0.95, 2.13)	0.344	1.21 (0.93, 1.62)	1.05 (0.80, 2.13)	0.771
HDL, *M* (Q1, Q3) (mmol/L)	1.10 (0.96, 1.26)	1.07 (0.94, 1.22)	0.435	1.05 (0.91, 1.26)	1.12 (1.03, 1.28)	0.084
LDL, *M* (Q1, Q3) (mmol/L)	2.69 (2.19, 3.23)	2.49 (2.01, 2.96)	0.002^**^	2.49 (1.94, 3.00)	2.56 (1.90, 2.84)	0.861
Hcy, *M* (Q1, Q3) (μmol/L)	10.3 (8.5, 12.7)	10.6 (8.2, 13.4)	0.651	11.1 (9.6, 13.8)	12.2 (10.4, 15.9)	0.097
BUN, M (Q1, Q3) (mmol/L)	5.80 (4.87, 6.90)	5.76 (4.65, 7.03)	0.855	6.90 (5.49, 9.10)	6.16 (4.92, 7.12)	0.077
Cr, M (Q1, Q3)(umol/l)	62 (53, 77)	60 (49, 78)	0.200	72 (60, 88)	67 (57, 83)	0.286
UA, *M* (Q1, Q3) (μmol/l)	309 (262, 370)	280 (233, 344)	<0.001^***^	351 (302, 436)	330 (280, 371)	0.019^*^
NIHSS score at admission, *M* (Q1, Q3)	2.0 (1.0, 4.0)	9.0 (5.0, 12.0)	<0.001^***^	2 (1, 5)	12 (9, 20)	<0.001^***^
NIHSS score at discharge, *M* (Q1, Q3)	1.0 (0.0, 2.3)	9.0 (6.0, 11.3)	<0.001^***^	1 (1, 3)	13 (8, 20)	<0.001^***^
mRS score at admission, *M* (Q1, Q3)	2.00 (1.00, 3.00)	4.00 (4.00, 5.00)	<0.001^***^	2.00 (1.50, 4.00)	5.00 (4.50, 5.00)	<0.001^***^
mRS score at discharge, *M* (Q1, Q3)	1.00 (0.00, 2.00)	4.00 (4.00, 4.00)	<0.001^***^	1.00 (1.00, 2.00)	5.00 (4.00, 5.00)	<0.001^***^
NIHSS score at admission			<0.001^***^			<0.001^***^
≤4	437 (76.4%)	30 (21.4%)		67 (70.5%)	3 (6.4%)	
>4	135 (23.6%)	110 (78.6%)		28 (29.5%)	44 (93.6%)	
TyG index	8.70 (8.35, 9.25)	9.03 (8.59, 9.57)	<0.001^***^	8.60 (8.25, 9.03)	8.65 (8.21, 9.48)	0.358
TyG index quartiles			<0.001^***^			0.027^*^
Q1	138 (24.1%)	26 (18.6%)		31 (32.6%)	17 (36.2%)	
Q2	162 (28.3%)	24 (17.1%)		20 (21.1%)	8 (17.0%)	
Q3	141 (24.7%)	37 (26.4%)		29 (30.5%)	6 (12.8%)	
Q4	131 (22.9%)	53 (37.9%)		15 (15.8%)	16 (34.0%)	

SBP, systolic blood pressure; DBP, diastolic blood pressure; Hcy, homocysteine; HbA1c, glycosylated hemoglobin; Cr, creatinine; Glu, glucose; TC, cholesterol; TG, triglyceride; HDL, high density lipoprotein; LDL, low-density lipoprotein; TOAST, trial of org 10172 in acute stroke treatment; CE, cardioembolic; LAA, large artery atherosclerotic; NIHSS, National Institute of Health Stroke Scale; mRS, modified Rankin Scale; TyG, triglyceride-glucose; *p < 0.05; **p < 0.01; ***p < 0.001.

### Interaction between TyG index and ischemic stroke subtypes (LAA/CE)

Formal interaction analysis was performed in the overall cohort to explore whether LAA and CE modified the association between the TyG index and poor functional outcome. As presented in Table 3, the multivariable logistic regression model adjusted for age, diastolic blood pressure, fasting blood glucose, total cholesterol, low-density lipoprotein cholesterol, uric acid, and admission NIHSS score showed no significant TyG index × stroke subtype interaction (OR = 0.777, 95%CI: 0.438–1.393, *p* = 0.391). Consistently, the likelihood ratio test confirmed that adding the interaction term did not improve model fit (*χ*^2^ = 0.730, *p* = 0.393).

**Table 3:** Results of TyG index and ischemic stroke subtypes (LAA/CE) interaction analysis and likelihood ratio test.

Interaction term	OR	95% CI	*p*-value
Continuous TyG index	—	—	—
TyG index × stroke subtype	0.777	0.438–1.393	0.391
Quartile TyG index	—	—	—
TyG Q2 × stroke subtype	1.006	0.245–4.043	0.993
TyG Q3 × stroke subtype	0.274	0.066–1.058	0.065
TyG Q4 × stroke subtype	0.586	0.166–2.095	0.408
Likelihood ratio test	—	—	—
Main-effect model vs. interaction model	—	—	0.393

All models were adjusted for variables with significant inter-group differences (*p* < 0.05), including age, diastolic blood pressure, fasting blood glucose, total cholesterol, low-density lipoprotein cholesterol, uric acid, and admission NIHSS score. TyG, triglyceride-glucose index; Q, quartile; OR, odds ratio; CI, confidence interval. Likelihood ratio test was performed to verify whether the inclusion of the TyG × stroke subtype interaction term improved model fitting.

Sensitivity analysis using quartile-stratified TyG categories further validated the results. No significant interactions were observed for TyG Q2 (*p* = 0.993), Q3 (*p* = 0.065), and Q4 (*p* = 0.408), with only a marginal non-significant trend for the Q3 interaction. Overall, no subtype-specific disparities in the prognostic effect of the TyG index on post-stroke poor outcome were identified between LAA and CE subtypes. The null association in the CE subgroup was likely due to limited statistical power caused by its smaller sample size, rather than a true differential effect.

### Association between the TyG index and the functional outcome at discharge of patients with LAA and CE

Multicollinearity was tested for the included variables, and VIF and tolerance values for each variable are depicted in Supplementary material 1. Variables with a VIF > 5 were excluded. The restricted cubic spline plot revealed a significant overall association rather than a nonlinear association between the TyG index and discharge functional outcomes in the LAA group after adjusting for gender, age, NIHSS score at admission, SBP, DBP, history of hypertension, diabetes, smoking, drinking, Hcy, BUN, Cr, and UA (*p*-nonlinear = 0.197, *p*-overall = 0.026; Figure 2A). However, no evidence of a significant nonlinear association between the TyG index and discharge-level functional outcome was found in the CE group (for nonlinearity, *p* = 0.089; Figure 2B). It should be noted that the sample size of the cardioembolic stroke subgroup was relatively small, with a limited number of poor-outcome events, which might result in insufficient statistical power. Therefore, a genuine association between the TyG index and functional outcomes in CE patients cannot be fully excluded.

**Figure 2:**
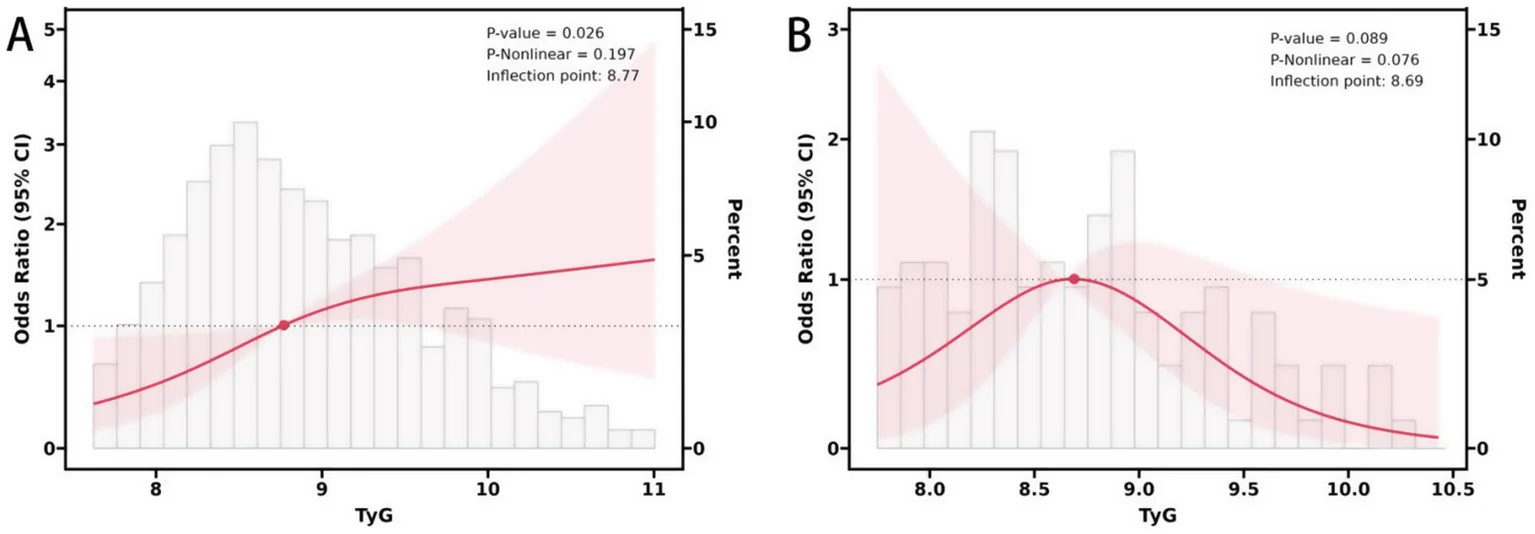
Restricted cubic spline analysis of the relationship between the TyG index and the functional outcome at discharge of LAA and CE strokes. **(A)** TyG index and LAA stroke functional outcome at discharge. **(B)** TyG index and CE stroke functional outcome at discharge. LAA, large artery atherosclerotic; CE, cardioembolic; TyG, triglyceride-glucose; CI, confidence interval.

Univariate logistic regression analysis of LAA and CE was performed using the selected baseline variables. The outcome variable was defined as an mRS score > 3 at discharge. Age, NIHSS score at admission, Glu, LDL, and TyG index were all statistically significant in the LAA group (*p* < 0.05; Figure 3A). Among the TyG index quartile groups, only the TyG-Q4 group was associated with the outcome variable (*p* < 0.05; Figure 3A). No significant association was found between the TyG index and the outcome variable in the CE group (*p* > 0.05; Figure 3B).

**Figure 3:**
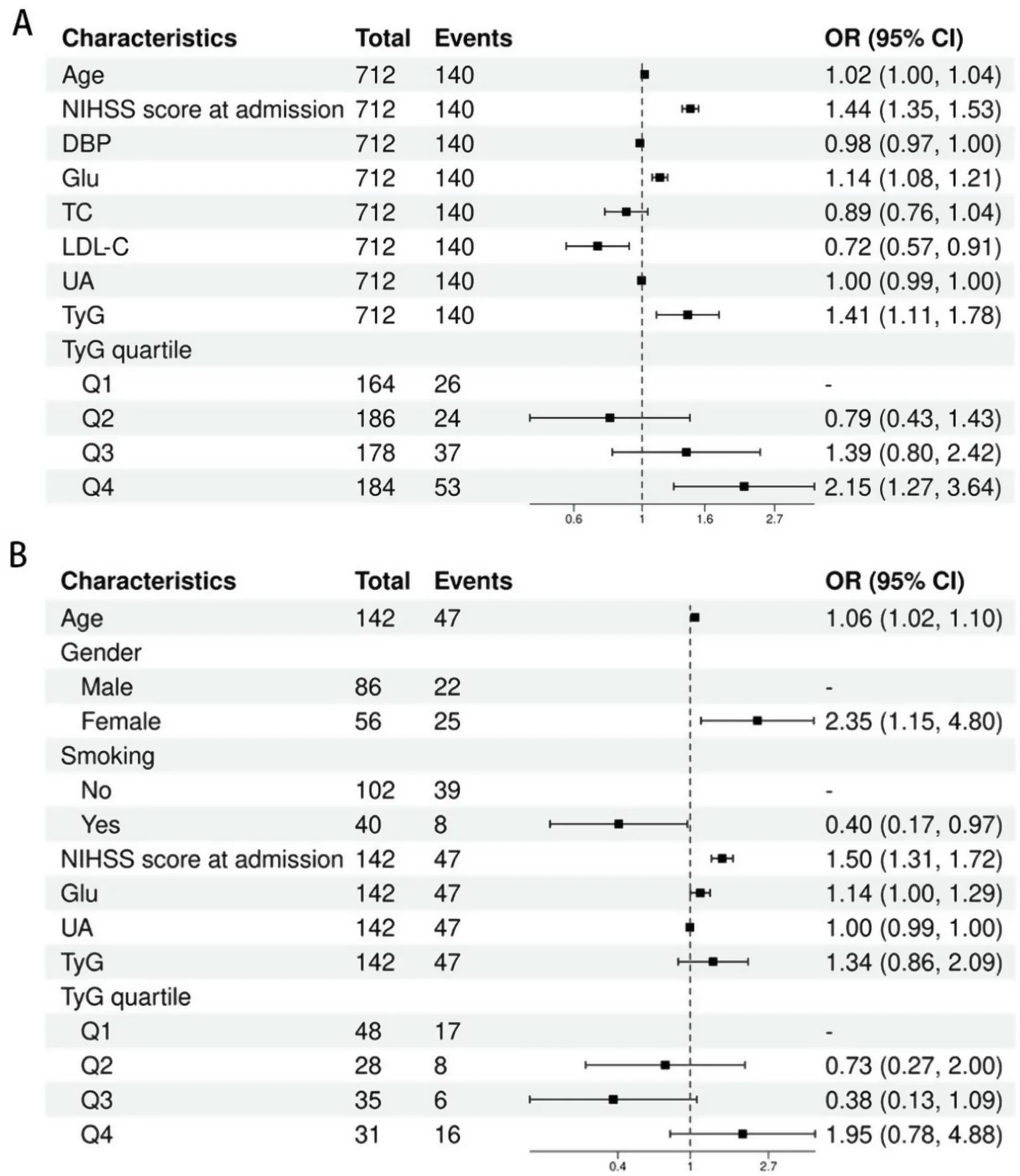
Univariate logistic regression analysis of the association between the TyG index and the functional outcome at discharge of LAA and CE. **(A)** TyG index and functional outcome at discharge of LAA; **(B)** TyG index and functional outcome at discharge of CE. DBP, diastolic blood pressure; Glu, glucose; TC, cholesterol; LDL, low-density lipoprotein; UA, uric acid; LAA, large artery atherosclerotic; CE, cardioembolic; NIHSS, National Institute of Health Stroke Scale; TyG, triglyceride-glucose; OR, odds ratio; CI, confidence interval.

Subsequently, multivariate logistic regression analysis of discharge-level functional outcome for LAA and CE patients was performed. In model 1 for LAA, the TyG-Q2 and TyG-Q3 groups were not associated with poor functional outcome at discharge compared with the TyG-Q1 group. However, the TyG-Q4 group was a risk factor for poor functional outcome at discharge in the LAA group (odds ratio (OR) = 2.63, 95% confidence interval (CI) = 1.52–4.55; Figure 4A). After adjusting for age, NIHSS score at admission, Glu, DBP, UA, and LDL, the TyG-Q4 group remained a risk factor for poor functional outcome at discharge in the LAA group (OR = 2.38, 95% CI = 1.03–5.51; Figure 4A). However, no association was found between the TyG index and discharge-level functional outcome in the CE group (Figure 4B).

**Figure 4:**
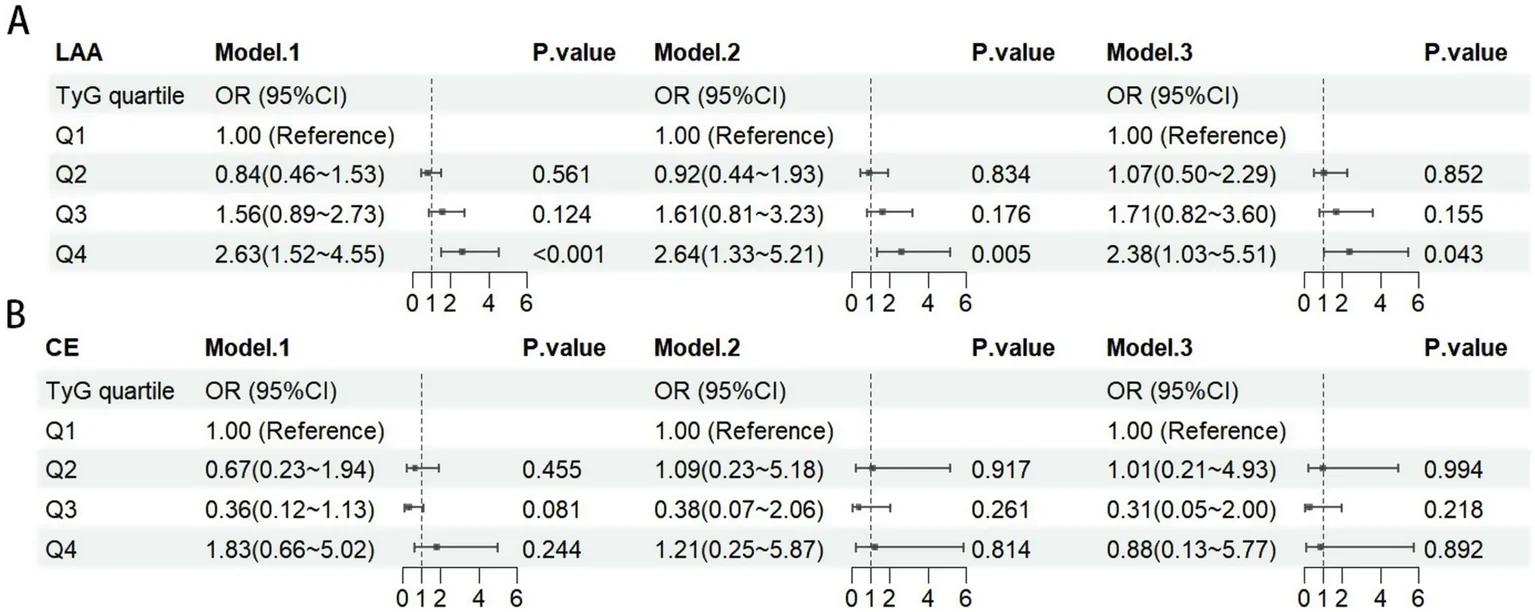
Multivariate logistic regression analysis of the relationship between the TyG index and the functional outcome at discharge of LAA and CE. **(A)** TyG index and functional outcome at discharge of LAA; **(B)** TyG index and functional outcome at discharge of CE; LAA, large artery atherosclerotic; CE, cardioembolic; TyG, triglyceride-glucose; OR, odds ratio; CI, confidence interval. **(A)** Model 1: adjusted for Age; Model 2: adjusted for Age and NIHSS score at admission; Model 3: adjusted for Age, NIHSS score at admission, Glu, DBP, UA, and LDL. **(B)** Model 1: adjusted for Age and Gender; Model 2: adjusted for Age, Gender, NIHSS score at admission, and Smoking; Model 3: adjusted for Age, Gender, NIHSS score at admission, Smoking, Glu, and UA.

### Predictive value of the TyG index in the functional outcome at discharge of LAA stroke

ROC curve analysis further evaluated the predictive performance of the TyG index for poor functional outcome at discharge in patients with LAA stroke. The standalone predictive ability of the TyG index was poor, with an unadjusted AUC of only 0.592, indicating limited discriminatory power when used alone. The markedly elevated AUC of 0.876 observed in the fully adjusted model was predominantly attributed to the incremental predictive contribution of traditional clinical and laboratory variables, including age, baseline NIHSS score, glucose, diastolic blood pressure, uric acid, and low-density lipoprotein, rather than the independent predictive effect of the TyG index itself. (Figure 5). As shown in Table 3, the predictive performance of the baseline clinical model and the combined model incorporating the TyG index was compared. The baseline clinical model yielded an AUC of 0.820 (95% CI: 0.78–0.86). The addition of the TyG index increased the AUC to 0.876 (95% CI: 0.84–0.91), indicating improved overall discriminatory capacity. However, as shown in Table 4, the incremental predictive value was not statistically significant. The continuous net reclassification improvement (NRI) was 0.002 (95% CI: −0.032 to 0.036, *p* = 0.901), and the integrated discrimination improvement (IDI) was 0.001 (95% CI: −0.002 to 0.005, *p* = 0.485). These results indicated that although the inclusion of the TyG index slightly improved the overall discrimination of the clinical model, it provided no significant additional risk reclassification and discriminatory power for predicting unfavorable functional outcomes in LAA stroke patients.

**Figure 5:**
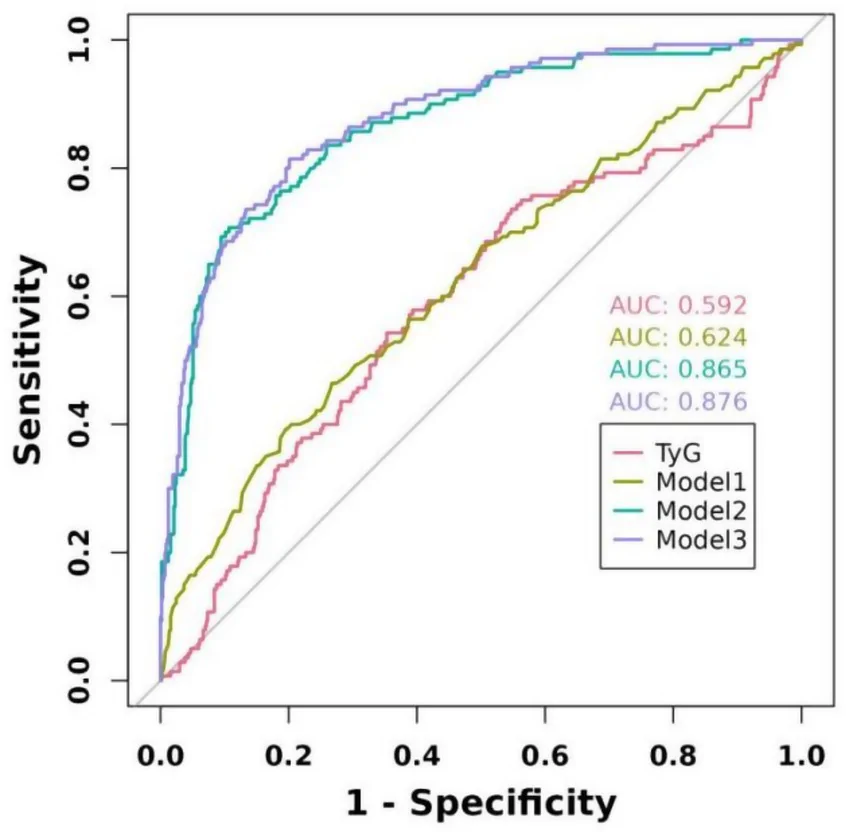
Receiver operating characteristic analysis of the predictive value of the TyG index for functional outcome at discharge of LAA. TyG, triglyceride-glucose; AUC, area under the curve. Model 1: adjusted for age; Model 2: adjusted for age and NIHSS score at admission; Model 3: adjusted for age, NIHSS score at admission, Glu, DBP, UA, and LDL.

**Table 4:** Model comparison for predicting unfavorable discharge functional outcomes.

Model comparison	AUC (95% CI)	NRI (95% CI)	*p*-value (NRI)	IDI (95% CI)	*p*-value (IDI)
Model 1 (baseline clinical model)	0.820 (0.78–0.86)	Reference	Reference	Reference	Reference
Model 2 (baseline model + TyG index)	0.876 (0.84–0.91)	0.002 (−0.032 to 0.036)	0.901	0.001 (−0.002 to 0.005)	0.485

AUC, area under the receiver-operating characteristic curve; NRI, net reclassification improvement; IDI, integrated discrimination improvement; CI, confidence interval. Model 1: conventional baseline clinical variables including age, NIHSS score at admission, Glu, DBP, UA, and LDL. Model 2: baseline clinical variables plus the triglyceride-glucose (TyG) index. Reference: Model 1 was taken as the reference for calculating NRI and IDI.

### Association between the TyG index and the functional outcome at discharge of LAA stroke in various subgroups

Subgroup analyses stratified by age, gender, admission NIHSS score, smoking, alcohol consumption, diabetes, and hypertension were conducted to evaluate whether clinical characteristics modified the prognostic association between the TyG index and unfavorable functional outcomes in patients with LAA stroke. All subgroup results are presented in Table 5. Subgroup-specific analyses showed that an elevated TyG index significantly predicted poor prognosis in patients aged ≥60 years (OR = 1.47, 95% CI = 1.12–1.92), those with higher stroke severity (NIHSS > 4; OR = 1.57, 95% CI = 1.13–2.18), female patients (OR = 1.48, 95% CI = 1.05–2.08), smokers (OR = 1.85, 95% CI = 1.11–3.06), drinkers (OR = 2.08, 95% CI = 1.13–3.83), and hypertensive patients (OR = 1.56, 95% CI = 1.18–2.05). These subgroup ORs represent stratum-specific associations of the TyG index with functional outcomes, rather than independent prognostic effects of the stratified clinical variables. Notably, formal interaction analyses confirmed no significant multiplicative interaction across all stratified variables (all *p*-for interaction > 0.05), indicating that the predictive value of the TyG index for unfavorable outcomes was stable and not substantially modified by these conventional clinical factors in the LAA stroke population.

**Table 5:** Subgroup analysis of the association between the TyG index and LAA functional outcome at discharge.

Subgroup	Layer	OR	95% CI	*p*-value	*p*-for interaction
Age	<60	1.44	(0.85, 2.44)	0.174	0.6586
	≥60	1.47	(1.12, 1.92)	0.005^**^	
Gender	Female	1.48	(1.05, 2.08)	0.024^*^	0.6733
	Male	1.31	(0.94, 1.81)	0.0674	
Admission NIHSS	≤4	1.03	(0.62, 1.69)	0.918	0.9357
	>4	1.57	(1.13, 2.18)	0.008^**^	
Smoking	No	1.30	(1.00, 1.70)	0.0052^**^	0.7954
	Yes	1.85	(1.11, 3.06)	0.017^*^	
Drinking	No	1.31	(1.02, 1.69)	0.038^*^	0.7317
	Yes	2.08	(1.13, 3.83)	0.018^*^	
Diabetes	No	1.44	(1.06, 1.98)	0.022^*^	0.7940
	Yes	1.34	(0.89, 2.02)	0.163	
Hypertension	No	1.28	(0.80, 2.06)	0.304	0.8640
	Yes	1.56	(1.18, 2.05)	0.002^**^	

OR, odds ratio; CI, confidence interval. Subgroup-specific ORs represent the association between the TyG index and unfavorable functional outcomes within each stratum, rather than the independent prognostic effect of the stratified clinical variable. *p*-for interaction denotes the *p*-value for multiplicative interaction testing between the TyG index and corresponding stratifying variable. *p < 0.05; **p < 0.01. All interaction *p*-values >0.05, indicating no significant effect modification by these clinical factors.

## Discussion

The present study analyzed data from patients with acute ischemic stroke receiving standardized treatment. The results indicate that the TyG index was significantly associated with poor functional outcome at discharge in LAA patients, suggesting that it is an independent risk factor for unfavorable discharge-level functional outcomes in LAA. However, no significant association was observed between the TyG index and functional outcome at discharge in CE patients. Notably, this negative finding should be interpreted with extreme caution, as the sample size of the CE subgroup was relatively small, with only 142 enrolled CE patients and fewer than fifty cases presenting with poor functional outcomes at discharge. The limited number of endpoint events substantially reduced the statistical power of the subgroup analysis, which means the non-significant association may merely reflect insufficient statistical efficiency rather than a genuine absence of an association between the TyG index and discharge-level functional outcome among patients with CE stroke. Therefore, we cannot definitively conclude that the TyG index has no predictive value for CE patients, and adequate large-sample studies are still required to verify the relationship between the TyG index and clinical outcomes in the CE population. These subtype-specific differences further reflect the distinct pathological mechanisms underlying LAA and CE stroke.

LAA is one of the major subtypes of ischemic stroke, characterized by cerebral artery stenosis or occlusion caused by atherosclerosis (6). The occurrence and progression of atherosclerosis are closely associated with lipid metabolism disorders, especially TGs and insulin resistance, which play a key role (17, 18). The TyG index, calculated by the product of fasting Glu and TG levels, reflects an individual’s insulin resistance and lipid metabolism status, making it an effective predictor of the occurrence and recurrence risk of atherosclerotic stroke (19). Beverly et al. found that insulin resistance triggered a series of metabolic abnormalities, including elevated TG levels, endothelial dysfunction, and increased inflammatory response (20). These metabolic abnormalities promote the formation and instability of atherosclerotic plaques, thereby increasing the likelihood of stroke (21). Patients with higher TyG indices usually have more severe atherosclerosis and larger plaque volume, leading to relatively worse post-stroke outcomes (22, 23).

The TyG index showed certain but limited predictive value for discharge functional outcomes of LAA stroke patients. This finding aligns with the well-established impact of insulin resistance on the progression of atherosclerosis (21). Insulin resistance is closely associated with the instability of atherosclerotic plaques in LAA patients. Metabolic abnormalities caused by insulin resistance increase local inflammation, leading to heightened activity of immune cells such as macrophages and T cells within the plaques, further exacerbating plaque instability (24, 25). Elevated TG and Glu levels accelerate the oxidation and glycation of LDL, which not only increases endothelial cell damage but also enhances macrophage lipid uptake, forming foam cells (17). Excessive accumulation of foam cells leads to necrosis within atherosclerotic plaques, resulting in thrombosis and triggering acute stroke. Additionally, insulin resistance affects vascular endothelial cell function, reducing nitric oxide production, further weakening the protective function of blood vessels and impairing vascular repair capacity (26). These biological mechanisms collectively help explain the unfavorable discharge-level functional outcomes observed in LAA patients with higher TyG index after stroke. Therefore, the TyG index may act as a useful metabolic biomarker for identifying LAA patients at higher risk of poor functional outcome at discharge under standardized treatment. Elevated TyG index reflects underlying insulin resistance and disturbed glycolipid metabolism in LAA patients. However, given the observational nature of our study, we cannot infer that aggressive metabolic interventions will reduce stroke recurrence or improve discharge-level functional status; such causal hypotheses require further prospective investigation (27).

Although the TyG index was identified as an independent predictor of poor functional prognosis in our multivariable analysis, the NRI and IDI analyses revealed no significant incremental predictive value when added to the base clinical model. This apparent discrepancy can be explained by the “ceiling effect” of the base model. Traditional clinical variables, particularly baseline stroke severity (NIHSS score), are overwhelmingly strong predictors of short-term functional outcomes in LAA stroke. Once these dominant clinical factors are accounted for, the marginal predictive contribution of a single metabolic biomarker is naturally limited. Therefore, rather than serving as a high-performance risk-stratification tool, the TyG index is best utilized as a supplementary metabolic indicator. It helps identify underlying insulin resistance in LAA patients, thereby guiding individualized glycolipid management and targeted metabolic interventions to improve long-term vascular health.

CE is primarily caused by thrombus formation in the heart, which embolizes cerebral arteries, with atrial fibrillation or other cardiac dysfunction being the most common causes (28, 29).

Since its primary etiology is hemodynamic abnormalities in the heart, the core pathological mechanisms of CE are unrelated to atherosclerosis, and thus lipid metabolism disorders and insulin resistance have less influence on its prognosis (29). Atrial fibrillation can lead to blood stasis in the heart, increasing the risk of thrombus formation, which, upon embolization, can block cerebral arteries and cause stroke (30). Therefore, the clinical management of CE focuses on anticoagulant therapy and cardiac function management rather than metabolic regulation. Our study results revealed no significant association between the TyG index and prognosis in CE patients, consistent with the pathological mechanisms of CE, as CE is primarily caused by thrombus formation in the heart rather than atherosclerotic cerebral artery occlusion. Insulin resistance and lipid metabolism disorders are not associated with the occurrence and recurrence of CE. Anticoagulation therapy is the core measure for preventing stroke recurrence in CE patients, particularly in atrial fibrillation patients, where long-term use of warfarin or direct oral anticoagulants effectively reduces the recurrence rate (31).

Compared with LAA, the prognosis of CE patients is more dependent on cardiac function management and adherence to anticoagulant medication rather than metabolic status, and thus the TyG index has no significant predictive value for the functional outcome at discharge of CE patients.

While conventional treatments such as antiplatelet, lipid-lowering, and antihypertensive drugs included in standardized stroke protocols effectively reduce vascular risk, it should be emphasized that the present retrospective observational study can only demonstrate statistical association rather than causal relationships. Therefore, we cannot conclude that therapeutic interventions targeting the TyG index will directly improve functional stroke outcomes. Although elevated TyG index reflects underlying insulin resistance and disturbed glycolipid metabolism, definite recommendations for routine off-label use of insulin-sensitizing agents or triglyceride-lowering pharmacotherapy cannot be derived solely from our current findings. Observational studies in acute ischemic stroke have highlighted the prognostic relevance of multiple endocrine and lipid-related laboratory parameters for survival and functional outcome at discharge, as well as the prognostic impact of atherosclerotic risk factors on cardiovascular events. For instance, Tatar et al. reported that atherogenic lipid-derived biomarkers could predict short-term mortality among ischemic-stroke patients, and cardiovascular functional parameters also exhibit hidden bidirectional links with acute ischemic-stroke pathophysiology, as described by Ozkan et al. (32, 33). In this context, monitoring of metabolic markers including the TyG index may serve as a risk-identification tool in clinical practice for LAA patients with elevated metabolic burden. Lifestyle modification and metabolic risk-factor optimization remain reasonable general clinical considerations; however, whether pharmacologically-driven improvement in TyG-related metabolic profiles translates into better stroke recovery requires further dedicated prospective or interventional research. Regular follow-up to monitor blood glucose and lipid profiles may still be reasonable for LAA patients with elevated TyG index, though clinical treatment adjustments should follow established stroke guidelines rather than being guided by the TyG index alone.

The management strategies for CE and LAA stroke differ significantly. Anticoagulant therapy and cardiac function monitoring constitute the core management approaches for CE patients, while metabolic indicators receive relatively less clinical emphasis. Therefore, although the TyG index shows predictive value for poor functional outcome at discharge in LAA patients, clinicians should prioritize electrocardiography, echocardiography, and anticoagulation-related adjustments for CE patients rather than focusing on alterations in insulin resistance or the TyG index.

Despite identifying the predictive value of the TyG index in LAA patients, this study has several limitations. First, the sample size was relatively small, particularly for CE patients, which may restrict our capacity to detect potential associations. Thus, large-scale studies are required to further validate these findings across diverse populations. Second, the primary functional outcome of this study was assessed using the mRS at hospital discharge, instead of the 90-day mRS which is widely accepted as the standard primary endpoint for stroke outcome research. Discharge timing varied substantially across participants and between the two participating centers, which may lead to inconsistent evaluation of neurological recovery and limit interpretation of patient functional status. This endpoint-selection bias may affect the stability and generalizability of the association between the TyG index and stroke functional outcomes. We chose discharge-based mRS mainly because long-term follow-up would incur high loss-to-follow-up rates in our cohort, where many patients reside in geographically remote areas. Third, we only captured functional status at hospital discharge; therefore, we cannot draw conclusions regarding long-term post-discharge recurrence or sustained functional recovery. Stroke is a chronic condition, and recurrence and functional rehabilitation may continue over multiple years. Longer follow-up durations are needed to explore the long-term clinical relevance of the TyG index, which cannot be addressed by the present discharge-only endpoint. Fourth, although several established risk factors were adjusted for in statistical models, uncollected variables including lifestyle characteristics and medication adherence may still introduce residual confounding. Future studies should collect more granular lifestyle and medication data to mitigate confounding effects. Further research should explore the performance of the TyG index across additional stroke subtypes. Given the observational nature of our study, interventional studies are required to test whether metabolic management can modify patient outcomes for individuals with elevated TyG index; we cannot confirm such causal benefits from our current dataset. Future work may integrate the TyG index alongside other metabolic or inflammatory biomarkers (e.g., C-reactive protein, insulin levels) to build multi-factor risk-stratification models, which may enable more comprehensive evaluation of metabolic risk and support individualized clinical decision-making for ischemic-stroke patients.

## Conclusion

In summary, this study demonstrates that the TyG index serves as an independent risk factor for poor functional outcome at discharge in patients with atherosclerotic stroke who received standardized treatment. Given that insulin resistance and lipid metabolism disorders critically contribute to the onset and progression of atherosclerotic stroke, the TyG index can be utilized to screen high-risk patients and assist in individualized clinical risk stratification. In contrast, no significant association was observed between the TyG index and discharge-level functional outcomes in patients with cardioembolic stroke. This subtype-specific discrepancy indicates that clinical management for cardioembolic stroke should prioritize anticoagulation therapy and cardiac function monitoring, rather than metabolic intervention targeting the TyG index.

## Data Availability

The original contributions presented in the study are included in the article/Supplementary material, further inquiries can be directed to the corresponding authors.
